# Neurofilament Light Chain as a Biomarker of Neuronal Damage in Children With Malaria

**DOI:** 10.1093/infdis/jiad373

**Published:** 2023-08-30

**Authors:** Núria Balanza, Caroline K Francis, Valerie M Crowley, Andrea M Weckman, Kathleen Zhong, Bàrbara Baro, Rosauro Varo, Quique Bassat, Kevin C Kain, Rosiglitazone Adjunctive Therapy for Severe Malaria in Children (ROSI) Study Group, Alfredo Mayor, Ana Rosa Manhiça, Anelsio Cossa, Antonio Sitoe, Campos Mucasse, Clara Erice, Crisóstomo Fonseca, Humberto Mucasse, Justina Bramugy, Lazaro Quimice, Lena Serghides, Marta Valente, Melissa Gladstone, Pio Vitorino, Rubao Bila, Sara Ajanovic, Yiovanna Derpsch

**Affiliations:** ISGlobal, Hospital Clinic–University of Barcelona, Barcelona, Spain; Sandra-Rotman Centre for Global Health, Toronto General Research Institute, University Health Network–Toronto General Hospital, Toronto, Ontario, Canada; Sandra-Rotman Centre for Global Health, Toronto General Research Institute, University Health Network–Toronto General Hospital, Toronto, Ontario, Canada; Sandra-Rotman Centre for Global Health, Toronto General Research Institute, University Health Network–Toronto General Hospital, Toronto, Ontario, Canada; Tropical Disease Unit, Division of Infectious Diseases, Department of Medicine, University of Toronto, Toronto, Ontario, Canada; Sandra-Rotman Centre for Global Health, Toronto General Research Institute, University Health Network–Toronto General Hospital, Toronto, Ontario, Canada; ISGlobal, Hospital Clinic–University of Barcelona, Barcelona, Spain; ISGlobal, Hospital Clinic–University of Barcelona, Barcelona, Spain; Centro de Investigação em Saúde de Manhiça, Maputo, Mozambique; ISGlobal, Hospital Clinic–University of Barcelona, Barcelona, Spain; Centro de Investigação em Saúde de Manhiça, Maputo, Mozambique; Institució Catalana de Recerca i Estudis Avançats, Barcelona, Spain; Pediatrics Department, Hospital Sant Joan de Déu–‌‌‌University of Barcelona, Barcelona, Spain; Consorcio de Investigación Biomédica en Red de Epidemiología y Salud Pública, Madrid, Spain; Sandra-Rotman Centre for Global Health, Toronto General Research Institute, University Health Network–Toronto General Hospital, Toronto, Ontario, Canada; Tropical Disease Unit, Division of Infectious Diseases, Department of Medicine, University of Toronto, Toronto, Ontario, Canada

**Keywords:** biomarker, brain injury, malaria, neurofilament light chain, neuronal damage

## Abstract

Malaria can cause brain injury. Neurofilament light chain (NfL) is a biomarker of neuronal damage. Here we examined longitudinal plasma NfL levels in children aged 1–12 years with uncomplicated and severe malaria from Mozambique. NfL levels were similar in all malaria cases at hospital admission. However, levels increased over time and the increment was significantly higher in severe malaria cases with neurological manifestations (ie, coma, impaired consciousness, or repeated seizures). NfL may be useful to identify and quantify brain injury in malaria.

## Teaser

Neurofilament light chain (NfL) is a biomarker of neuronal damage. We quantified plasma NfL in children with severe and uncomplicated malaria. Levels were similar at admission but increased over time. The increment was significantly higher in cases with neurological manifestations.

Malaria is a leading cause of neurodisability in African children. Previous studies have linked malaria, and especially cerebral malaria, to long-term neurological deficits, cognitive impairments, and behavioral alterations in surviving children [1]. While the underlying mechanisms are incompletely understood, it has been proposed that malaria-induced injury to neurons and glial cells results in neurological sequelae [2]. Postmortem immunohistochemistry studies in fatal malaria have reported neuropathological features including axonal and myelin damage [3]. Nevertheless, current tools to evaluate brain injury or sequelae (ie, neuroimaging, electroencephalograms, or neurocognitive tests) are limited in malaria-endemic areas, hindering efforts to understand its pathobiology, manage patients accordingly, and evaluate putative neuroprotective interventions.

Neurofilament light chain (NfL) is a neuron-specific protein. It is located in the neuronal cytoplasm and is highly expressed in large, myelinated axons. Low levels of NfL are constantly released from neurons into the extracellular space and ultimately reach the cerebrospinal fluid (CSF) and blood. However, circulating NfL levels increase when axonal injury occurs due to inflammatory, neurodegenerative, traumatic, or vascular injury [4, 5]. NfL has been extensively studied in neurodegenerative diseases and traumatic brain injury [4, 5]. More recently, it has been measured in different infectious diseases (eg, sepsis, pneumonia, or meningitis), where NfL was associated with neurological signs and symptoms, brain damage, neurological sequelae, and unfavorable outcomes [6–10]. Here we tested the hypothesis that plasma NfL levels function as a biomarker of neuronal damage in children with malaria.

## METHODS

### Study Design

This study was conducted at the Manhiça District Hospital, in southern Mozambique. Children aged 1–12 years with malaria were enrolled in a randomized controlled trial of adjunctive therapy with rosiglitazone. The safety and tolerability of rosiglitazone were first assessed in uncomplicated malaria (UM) cases between February and March 2016 [11]. From March 2016 to December 2019, efficacy was studied in severe malaria (SM) cases (ClinicalTrials.gov identifier: NCT02694874). Children were included in both study phases if they had a positive malaria diagnostic test (blood smear or histidine-rich protein 2/*Plasmodium* lactate dehydrogenase rapid diagnostic test) and confirmation of >2500 parasites/µL on thick smears. SM was defined as having repeated seizures (≥2 in the preceding 24 hours), prostration, impaired consciousness (Blantyre Coma Scale score <5 or Glasgow Coma Scale score <15), respiratory distress (sustained nasal flaring, deep breathing, or subcostal retractions), hypoglycemia (glucose <2.5 mmol/L), hyperlactatemia (lactate >5 mmol/L), or requiring hospitalization and parenteral treatment based on physician assessment. Children with known underlying illness (ie, neurological or neurodegenerative disorders; cardiac, renal, or hepatic disease; diabetes; epilepsy; or cerebral palsy), presenting solely with severe anemia (considered neither UM nor SM cases), or human immunodeficiency virus type 1 positive were excluded. Apart from the intervention, all children received the Mozambican standard of care for UM (artemether-lumefantrine) or SM (intravenous artesunate).

### Plasma NfL Quantification

Venous blood was collected in ethylenediaminetetraacetic acid–coated tubes and centrifugated, and the derived plasma was isolated and stored at −80°C until shipment to the University Health Network, Canada. NfL concentrations were determined using a commercial Simple Plex NfL assay kit (ProteinSimple, San Jose, California) on the ELLA microfluidic platform. NfL was quantified in samples from all participants obtained at admission and at 12, 36, 60, and 84 hours. For SM cases, samples from the follow-up visits at 96 hours and day 14 were also included. Plasma was diluted at 1:2. ELLA provides triplicate data for each sample and those with a coefficient of variation >20% were excluded. Concentrations under the dynamic range were assigned a value of half of the lowest detection limit. Procedures were performed according to the manufacturer's instructions and blinded to clinical data.

### Statistical Methods

Data were analyzed using Stata version 16.0 (StataCorp, College Station, Texas) and R version 4.0.3 (R Core Team, Vienna, Austria) software. The kinetics of NfL were assessed using linear mixed-effects (LME) models with intercepts modeled for each subject as random effects. An interaction term between each variable of interest (severity group, coma, impaired consciousness, or repeated seizures) and time postadmission was included in LME models. All models also incorporated child age, child sex, and treatment arm (placebo vs rosiglitazone) as fixed effects. Biomarker data were log-transformed for inclusion in LME models. Wald tests were used to assess NfL differences at hospital admission by severity group, interaction effects, and NfL slopes in each group. A 2-sided *P* value of <.05 was considered statistically significant.

### Ethical Approval

This study was reviewed and approved by the Mozambican National Bioethics Committee (reference number 230/CNBS/15), the pharmaceutical department of the Mozambican Ministry of Health (reference number 374/380/DF2016), the Clinical Research Ethics Committee of the Hospital Clinic, Barcelona, Spain (reference number HCB/2015/0981), and the University Health Network Research Ethics Committee, Toronto, Canada (reference number 15-9013-AE). All research was conducted according to the principles expressed in the Declaration of Helsinki. After a detailed explanation of the study, parents or legal guardians provided written informed consent and children aged >8 years gave verbal assent.

## RESULTS

A total of 167 malaria-positive children (30 with UM and 137 with SM) had at least 1 sample available for NfL quantification and were included in this study. This represents 100% (30/30) of recruited UM cases and 76.1% (137/180) of recruited SM cases. However, the distribution of demographic and clinical features was similar between SM children with and without NfL assessment. Table 1 presents the characteristics of the study cohort. At hospital admission, 59.9% (82/137) of children with SM displayed neurological manifestations: 15 had coma, 40 had impaired consciousness, and 69 had a history of ≥2 seizures in the preceding 24 hours. The overlap between these neurological manifestations is depicted in Supplementary Figure 1.

**Table 1.** Study Population Characteristics

Characteristic	UM (n = 30)	SM Without Neurological Manifestations^a^ (n = 55)	SM With Neurological Manifestations^a^ (n = 82)
Demographics			
Female sex	18 (60.0)	25 (45.5)	41 (50.0)
Age, y, median (IQR)	7.1 (4.7–9.2)	3.2 (1.9–5.1)	3.0 (2.2–4.1)
Baseline clinical characteristics			
Temperature, °C, median (IQR)	36.9 (36.3–38.1)	38.0 (37.2–39.1)	38.5 (37.7–39.5)
Respiratory distress^b^	0 (0)	11 (20.0)	18 (22.0)
History of ≥2 seizures in the preceding 24 h	0 (0)	0 (0)	69 (84.1)
Prostration	0 (0)	40 (72.7)	67 (81.7)
Impaired consciousness (BCS <5 or GCS <15)	0 (0)	0 (0)	40 (48.8)
Coma (BCS <3 or GCS <9)	0 (0)	0 (0)	15 (18.3)
Weight-for-age *z* score^c^, mean (SD)	−0.9 (1.0)	−0.8 (0.9)	−0.9 (1.2)
Baseline laboratory findings			
Hypoglycemia (glucose <2.5 mmol/L)	0 (0)	1 (1.8)	6 (7.3)
Hyperlactatemia (lactate >5 mmol/L)	0 (0)	14 (25.5)	17 (20.7)
Severe anemia (hemoglobin ≤5 g/dL)	0 (0)	5 (9.1)	4 (4.9)
Treatment arm			
Placebo	10 (33.3)	43 (78.2)	44 (53.7)
Rosiglitazone	20 (66.7)	12 (21.8)	38 (46.3)
Outcome			
Discharged alive	30 (100)	55 (100)	79 (96.3)
Transferred	0 (0)	0 (0)	1 (1.2)
Death	0 (0)	0 (0)	2 (2.4)

Data are presented as No. (%) unless otherwise indicated.

Abbreviations: BCS, Blantyre Coma Scale score; GCS, Glasgow Coma Scale score; IQR, interquartile range; SD, standard deviation; SM, severe malaria; UM, uncomplicated malaria.

^a^At admission, having coma, impaired consciousness, or a history of ≥2 seizures in the preceding 24 hours.

^b^Sustained nasal flaring, deep breathing, or subcostal retractions.

^c^Calculated using the LMS method and the 2000 US Centers for Disease Control and Prevention Growth Reference data.

**Figure 1.**
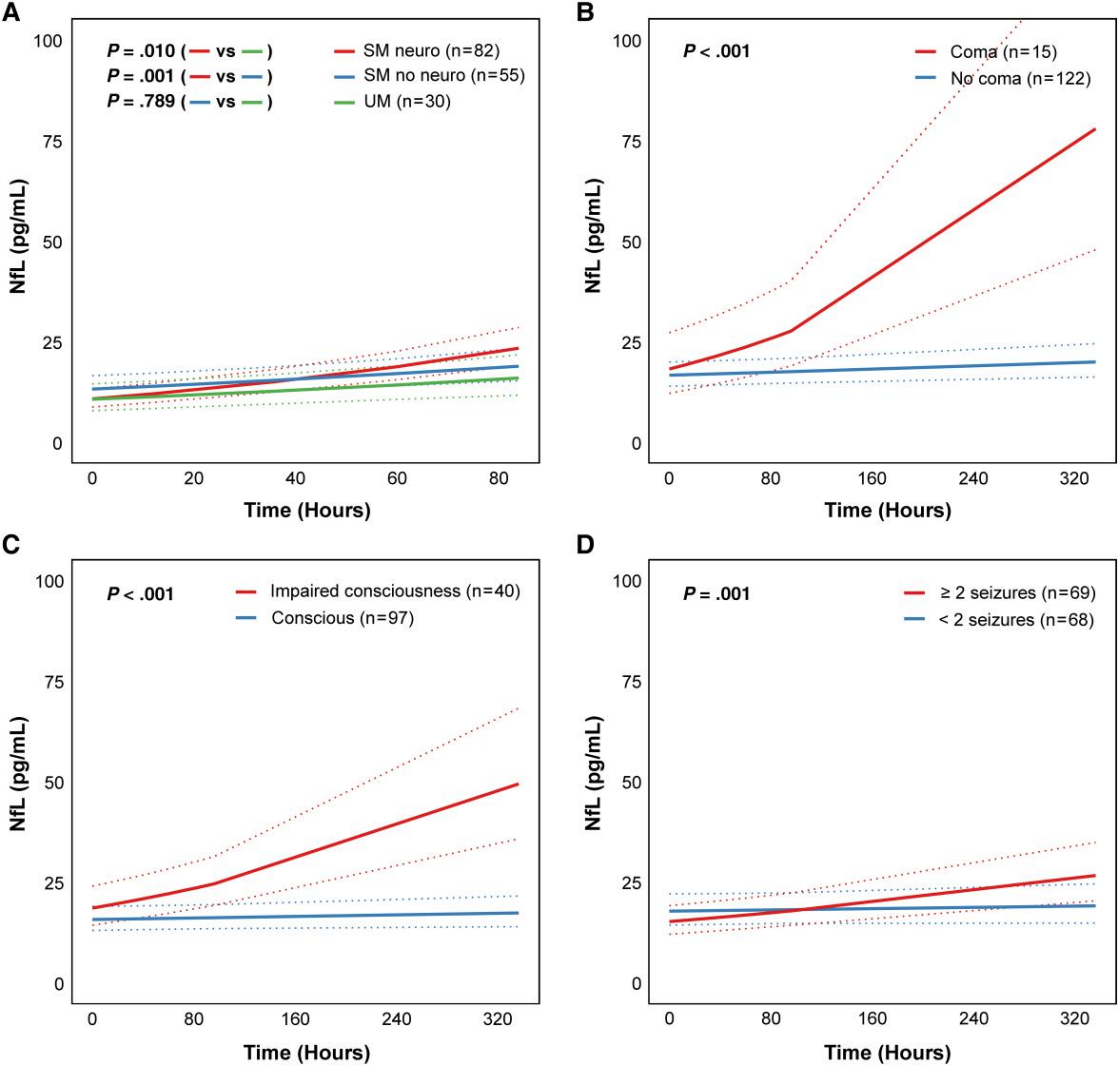
Plasma neurofilament light chain (NfL) concentrations in children with malaria. Longitudinal differences in concentrations of plasma NfL from admission to 84 hours by severity group (uncomplicated malaria [UM], severe malaria [SM] without neurological manifestations, or SM with neurological manifestations) (*A*) or from admission to day 14 by presence of each clinical neurological trait in children with SM (*B–D*). Longitudinal regression lines depict linear mixed-effects (LME) model–predicted, back-transformed values and 95% confidence intervals of NfL over time stratified by severity group (*A*) or clinical trait (*B–D*), while holding other fixed effects constant. All LME models included time postadmission, child sex, child age, and treatment arm (rosiglitazone vs placebo) as fixed effects, and a by-participant intercept as a random effect. An interaction term between severity group (*A*) or each clinical trait (*B–D*) and time postadmission was included in each model to account for variation in NfL over time. *P* values overlaid on graphs indicate the statistical significance of the interaction term assessed using a Wald test. A total of 30 children had UM, 55 had SM without neurological manifestations, and 82 had SM with neurological manifestations (15 with coma, 40 with impaired consciousness, and 69 with a history of ≥2 seizures in the preceding 24 hours). For the selected timepoints, 71.1% (788/1109) of samples were available for NfL quantification.

After adjusting for age, sex, and treatment arm, NfL levels at hospital admission were not significantly different between UM cases, SM cases without neurological manifestations, and SM cases with neurological manifestations (all *P* > .050). NfL concentrations increased significantly in all children over 84 hours (*P* = .001 for UM, *P* < .001 for both SM groups). The adjusted increase in NfL over 84 hours was higher in SM cases with neurological manifestations compared to the other 2 groups (*P* = .010, *P* = .001), but NfL longitudinal dynamics did not differ between SM without neurological manifestations and UM (*P* = .789) (Figure 1*A*). Among SM cases, the adjusted increase in NfL over 14 days was higher in children presenting each of the neurological manifestations. There was a significant interaction between time postadmission and coma (*P* < .001), impaired consciousness (*P* < .001), and repeated seizures (*P* = .001) (Figure 1*B–D*). In all models, NfL levels were negatively associated with age, but NfL levels were not associated with sex or treatment received from the clinical trial.

## DISCUSSION

In this study we longitudinally evaluated NfL, a biomarker of neuronal damage, in a cohort of malaria patients. The only previous report of NfL quantification in malaria was a case report of a traveler presenting with postmalaria acute motor axonal neuropathy [12]. Another study determined NfL levels in a mouse model of experimental cerebral malaria, where levels were elevated in mice with acute disease, but did not distinguish fatal from reversible brain edema [13]. Here we report that NfL levels at hospital admission were similar in all malaria cases irrespective of severity or clinical neurological traits. NfL levels increased over time in all children with malaria infection, suggesting some degree of subclinical neuronal damage in both UM cases and SM cases without overt neurological symptomatology [1]. However, the increase was more pronounced in SM cases being admitted with neurological manifestations. Thus, there was a delayed increase in NfL and it continued to rise through day 14.

When neuronal damage occurs at a specific and known timepoint, such as in traumatic brain injury, NfL levels in blood and CSF increase over a few days [4]. A progressive increase in NfL levels has also been reported in infectious diseases with neurological involvement, similar to the trend observed in our study [8–10]. The dynamics of blood NfL in pediatric malaria seem to differ from those of other brain injury biomarkers such as tau [14]. In fact, differences between these biomarkers were previously noted by authors working on traumatic brain injury [15]. Future studies are necessary to compare NfL to other proposed biomarkers of neuronal damage in malaria, with respect to kinetics and potential clinical utility.

The limited data available on NfL in healthy populations describe variations in levels according to age. Younger children have higher NfL levels and these decrease with age until reaching the lowest level at 10–15 years old [5]. NfL then increases up to late adulthood and rises steeply afterward, probably due to structural and metabolic changes that occur with aging [4, 5]. Consequently, it is important to consider age with special caution when studying NfL changes in pathological conditions. Here we noticed that age was an important confounder and adjusted all results for age. Nevertheless, we lacked NfL data in age-matched healthy community controls to define population-specific normal ranges for comparison.

This study benefits from the inclusion of multiple sample timepoints collected from a well-characterized cohort. However, it has limitations including a limited number of UM cases and a follow-up period of 84 hours (UM) or 14 days (SM) postenrollment. Further testing of later times is required to better understand NfL kinetics and to determine when levels plateau or normalize. Moreover, a larger sample size would be necessary to analyze possible interactions between neurological manifestations impacting NfL longitudinal dynamics. We did not associate NfL changes with neuroimaging, electroencephalographic results, or long-term neurocognitive testing. Children were participants in a randomized controlled trial, but there were no significant differences in NfL levels between treatment arms and results were adjusted for treatment received. We cannot exclude the possibility that other factors besides age and sex could affect NfL levels [5, 6]. Larger studies are needed to elucidate how additional factors might influence NfL levels, including underlying illnesses that were excluded as per study inclusion criteria, and how this impacts the interpretation of NfL levels as indicative of malaria-associated neuronal damage. Last, we lacked detailed information on previous episodes of SM or other infections that could contribute to cumulative neuronal damage and affect NfL levels during a given malaria episode.

Additional studies in malaria with larger cohorts and long-term outcomes are required. NfL could be useful to predict persistent neurocognitive dysfunction, allowing targeted follow-up and cognitive rehabilitation to children with the highest risk, as well as to serve as a surrogate marker for interventions designed to reduce neurocognitive impairment. The new immunoassays Simoa and ELLA have increased sensitivity and allow NfL quantification in blood, avoiding the need for invasive lumbar punctures to obtain CSF (where NfL levels are estimated to be ∼40 times higher) and opening the door to easily incorporate NfL quantification in studies conducted in malaria-endemic settings [4].

Our results support plasma NfL as a potential biomarker of neuronal damage in pediatric malaria. However, these findings indicate that NfL is not an early indicator, given its gradual increase after neurological manifestations appear. Measurement of NfL levels during malaria infection may enhance the identification and quantification of brain injury.

## Supplementary Data

Supplementary materials are available at *The Journal of Infectious Diseases* online. Consisting of data provided by the authors to benefit the reader, the posted materials are not copyedited and are the sole responsibility of the authors, so questions or comments should be addressed to the corresponding author.

## Supplementary Material

jiad373_Supplementary_Data

## Notes

***Acknowledgments.*** We are grateful to the patients and their families for participation in this study. We thank the nurses, field assistants, laboratory technicians, and other hospital staff who cared for the patients and contributed to study data collection. We also thank Dan Ouchi for statistical advice during this study.

***Author contributions.*** K. C. K. and V. M. C. conceived the study. N. B., C. K. F., V. M. C., A. M. W., B. B., R. V., Q. B., and K. C. K. contributed to study design. R. V. and V. M. C. were involved in study implementation. R. V. acquired clinical data. C. K. F., V. M. C., and K. Z. acquired the biomarker data. N. B., V. M. C., and R. V. were involved in data management. N. B. and A. M. W. conducted the statistical analysis. N. B. and C. K. F. drafted the manuscript. N. B., C. K. F., V. M. C., A. M. W., B. B., R. V., Q. B., and K. C. K. interpreted the data and critically revised the manuscript. All authors read and approved the final manuscript.

***ROSI Study Group.*** Alfredo Mayor, Ana Rosa Manhiça, Anelsio Cossa, Antonio Sitoe, Campos Mucasse, Clara Erice, Crisóstomo Fonseca, Humberto Mucasse, Justina Bramugy, Lazaro Quimice, Lena Serghides, Marta Valente, Melissa Gladstone, Pio Vitorino, Rubao Bila, Sara Ajanovic, and Yiovanna Derpsch.

***Data availability.*** The data that support the findings of this study are available from the corresponding author upon reasonable request.

***Disclaimer.*** The funding bodies played no role in study design; collection, analysis, or interpretation of the data; writing of the report; or the decision to submit the manuscript for publication.

***Financial support*.** This work was supported by the Canadian Institutes of Health Research Foundation grant FDN-148439 (to K. C. K.); a Canada Research Chair in Molecular Parasitology (to K. C. K.); and the Thomas Mather Fund (to K. C. K.). ISGlobal receives support from the grant CEX2018-000806-S funded by MCIN/AEI/10.13039/501100011033, and support from the Generalitat de Catalunya through the CERCA program. This research is part of ISGlobal's Program on the Molecular Mechanisms of Malaria, which is partially supported by the Fundación Ramón Areces. Centro de Investigação em Saúde de Manhiça is supported by the Government of Mozambique and the Spanish Agency for International Development. N. B. is supported by an FPU predoctoral fellowship from the Spanish Ministry of Universities (FPU18/04260). B. B. is a Beatriu de Pinos postdoctoral fellow granted by the Government of Catalonia’s Secretariat for Universities and Research and by the Marie Sklodowska-Curie Actions COFUND Program (BP3, 801370). R. V. had a fellowship from the program Río Hortega of the Instituto de Salud Carlos III (CM16/00024) during the conduct of the study. Funding to pay the Open Access publication charges for this article was provided by the grant CEX2018-000806-S funded by MCIN/AEI/10.13039/501100011033.
