# Supplementary material for: Neurofilament Light Chain as a Biomarker of Neuronal Damage in Children With Malaria
Source: J Infect Dis. 2023 Aug 30;229(1):183–8. doi: 10.1093/infdis/jiad373 (PMC10786245; doi:10.1093/infdis/jiad373)

**Neurofilament light chain as a biomarker of neuronal damage in children with malaria**

**Núria Balanza, Caroline K. Francis, Valerie M. Crowley, Andrea M. Weckman, Kathleen Zhong, Bàrbara Baro, Rosauro Varo, Quique Bassat, Kevin C. Kain; for the ROSI Study Group**

**SUPPLEMENTARY MATERIAL**

Supplementary Figure 1. Euler diagram showing the overlap between neurological manifestations in children with severe malaria


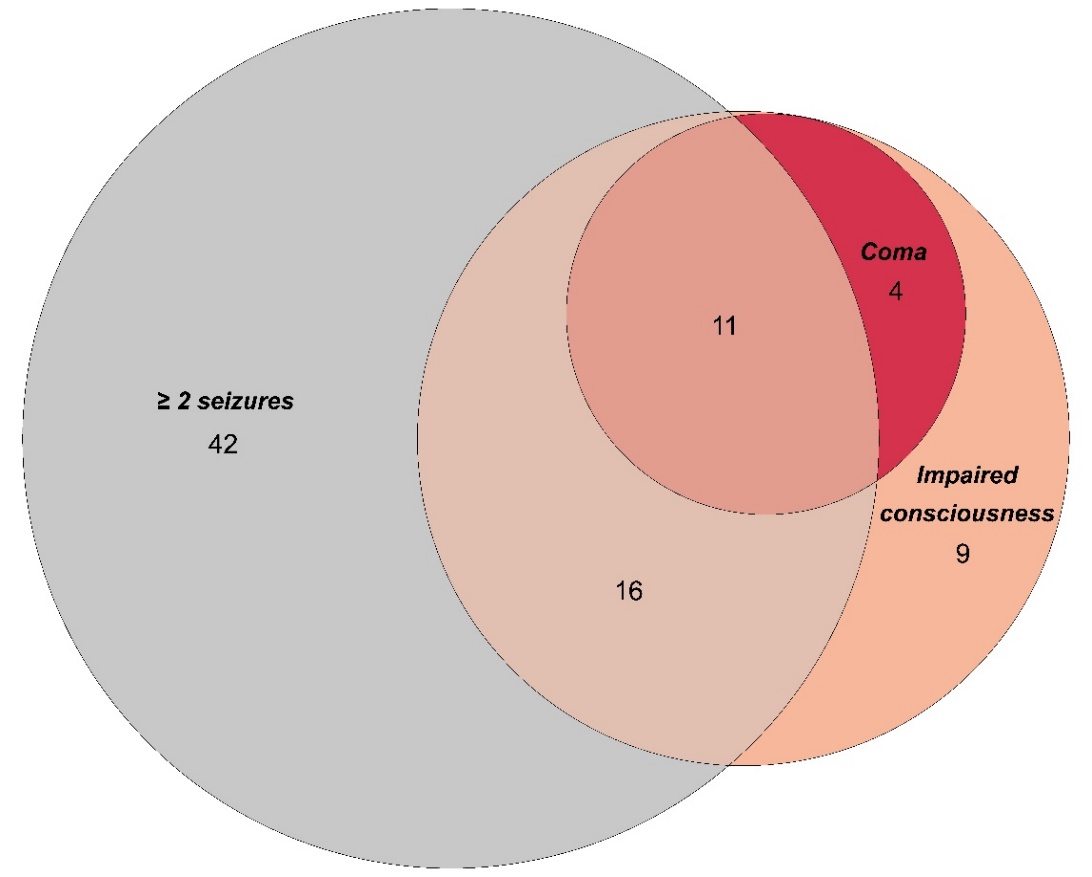

Supplement: jiad373_Supplementary_Data [file jiad373_supplementary_data.docx]
